# Stress Fracture of Bilateral Neck of Femur in a Healthy Non-athletic Young Adult – A Case Report With Review of Literature

**DOI:** 10.7759/cureus.11193

**Published:** 2020-10-27

**Authors:** Divesh Jalan, Khushwant Singh Rathore, Abhay Elhence, Sandeep Kumar Yadav, Deepak Kumar Maley

**Affiliations:** 1 Orthopaedics, Central Institute of Orthopaedics, Vardhman Mahavir Medical College & Safdarjung Hospital, New Delhi, IND; 2 Orthopaedics, National Institute of Medical Sciences and Research, Jaipur, IND; 3 Orthopaedics, All India Institute of Medical Sciences, Jodhpur, IND; 4 Trauma and Emergency Orthopaedics, All India Institute of Medical Sciences, Raipur, IND; 5 Orthopaedics, MediCiti Institute of Medical Sciences, Hyderabad, IND

**Keywords:** stress fracture, neck of femur, dynamic hip screw

## Abstract

Stress fractures of femoral neck are rare injuries and are usually seen in military recruits, marathon runners, and elderly with osteoporosis, renal rickets, steroid abuse, and metabolic bone diseases. Bilateral involvement of femoral neck in a healthy, non-athletic young adult is an extremely rare entity. We report one such case of a 36-year-old male who presented with bilateral groin pain for last three months. He had a history of excessive running prior to the onset of pain. The investigations confirmed bilateral stress fracture of the femoral neck, and the patient was operated with dynamic hip screw (DHS) on both sides. He returned to his routine activities in six months and at latest follow-up after two years; he is asymptomatic and has full function at both hips. This report highlights a rare cause of bilateral groin pain in a young adult, which requires early diagnosis and treatment to prevent complications.

## Introduction

Stress fractures of femoral neck are rare injuries, and bilateral involvement is even rarer. It was first reported by Blecher in 1905 [1]. It generally occurs in military recruits, marathon runners, and elderly with osteoporosis, renal rickets, steroid abuse, and metabolic bone diseases. These patients usually present with chronic groin pain without history of any significant trauma [2]. As a result, these fractures are very commonly missed unless a thorough radiographic evaluation is advised. A very high index of suspicion is required to diagnose these stress fractures. The literature is sparse regarding bilateral stress fractures in non-athletes [3]. We, therefore, report a rare case of stress fracture of bilateral neck of femur in a young healthy adult.

## Case presentation

A 36-year-old male presented with complaints of pain in both hips and difficulty in walking since three months. The pain initially started in the left hip and then involved the right hip after two weeks. It was insidious in onset, dull aching, and gradually progressive. At presentation to us, he was unable to bear weight on both sides. He was a non-smoker and occasionally consumed alcohol (60 ml/day, six to seven times in a month). A detailed history excluded any previous illness, chronic drug intake except for 10 intramuscular injections of nandrolone decanoate (DD®-50) over last three months. There was no history of trauma, epilepsy, or renal disease. There was a history of excessive physical activity in the form of running for two months prior to the onset of pain. He started initially with 5 kms and then gradually increased his running distance to 10 kms over a month. His physical examination revealed tenderness over bilateral hip joints (left > right). He had painful but full range of movements at both the hips. There was no shortening or deformity, and neurovascular examination was unremarkable. His routine blood tests including inflammatory markers, such as erythrocyte sedimentation rate (ESR) and C-reactive protein (CRP), and calcium profile including serum calcium, phosphorus, alkaline phosphatase, and Vitamin D levels were normal. The levels of parathyroid and thyroid hormones were also within normal limits. The T and Z scores on dual-energy x-ray absorptiometry (DEXA) scan at hip and spine were within normal range. 

**Figure 1:**
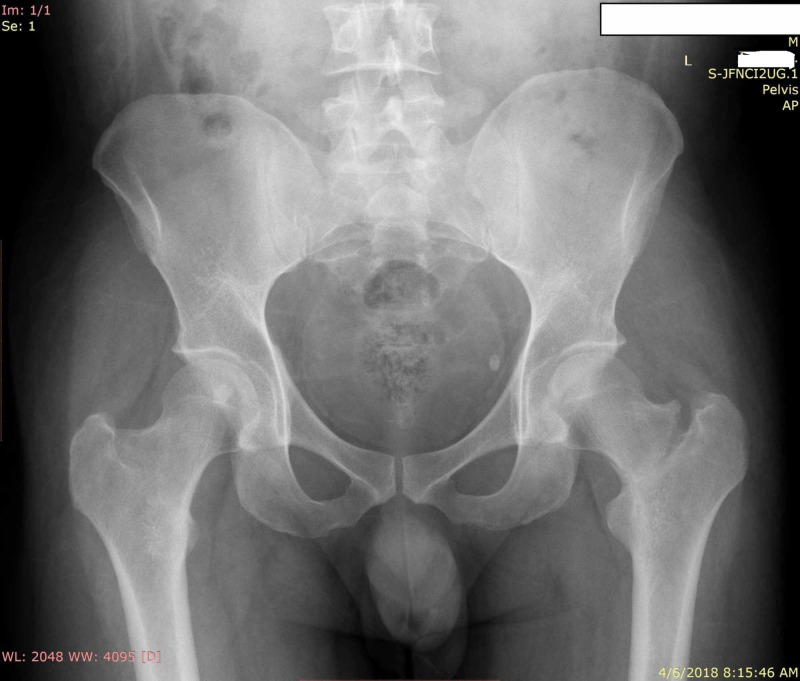
X-ray of the pelvis with both hips showing displaced basicervical fracture of left neck of femur.

His radiographs revealed a displaced basicervical neck of femur fracture on the left side, while the right hip appeared normal (Figure 1).

**Figure 2:**
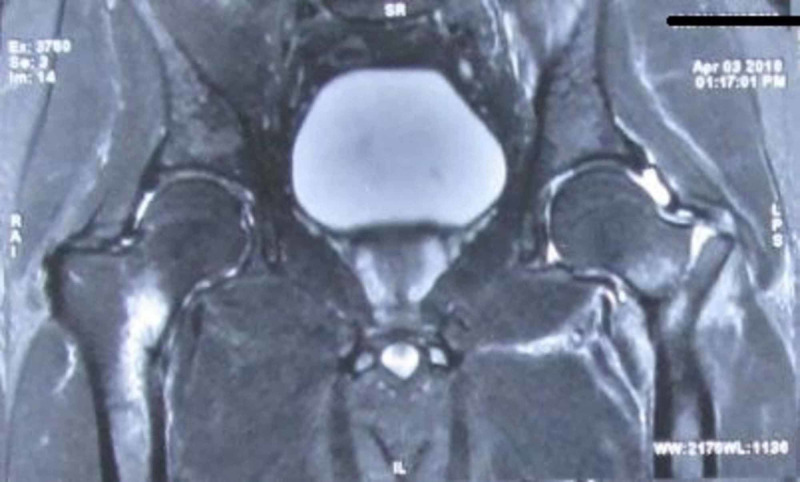
MRI showing displaced basicervical neck of femur fracture on the left side and incomplete neck of femur fracture on the right side. MRI: Magnetic resonance imaging.

Advanced imaging with MRI of the pelvis with both hips revealed displaced basicervical neck of femur fracture on the left side and incomplete fracture on the right side also (Figure 2).

**Figure 3:**
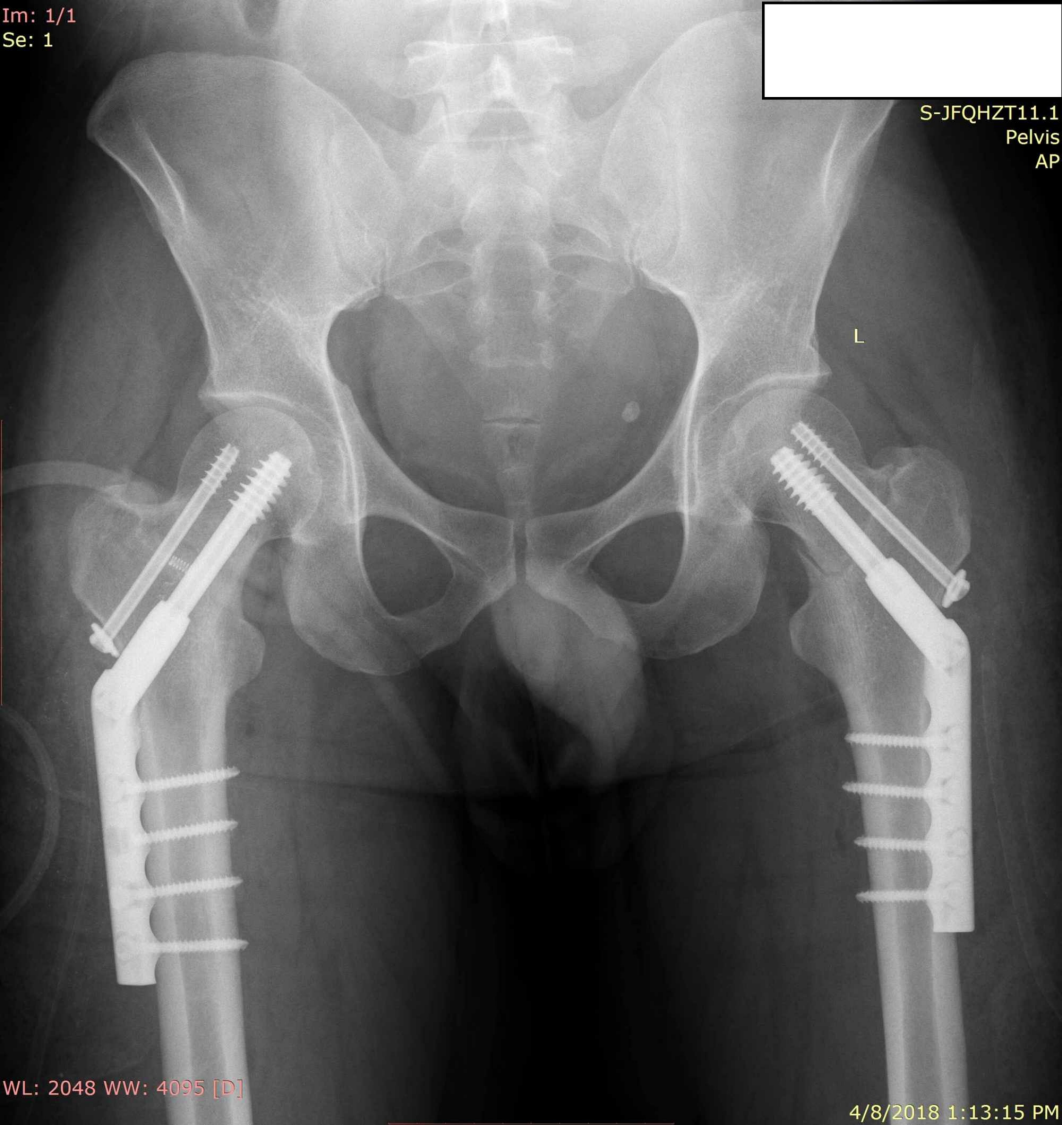
Immediate post-operative x-ray showing internal fixation using dynamic hip screw on both sides.

Thus, the diagnosis of bilateral stress fracture of femoral neck was made, and the patient was admitted for surgical stabilization. After informed consent, the patient was operated with closed reduction and internal fixation with dynamic hip screw (DHS) (Titanium, SMPL®) on both sides (Figure 3).

**Figure 4:**
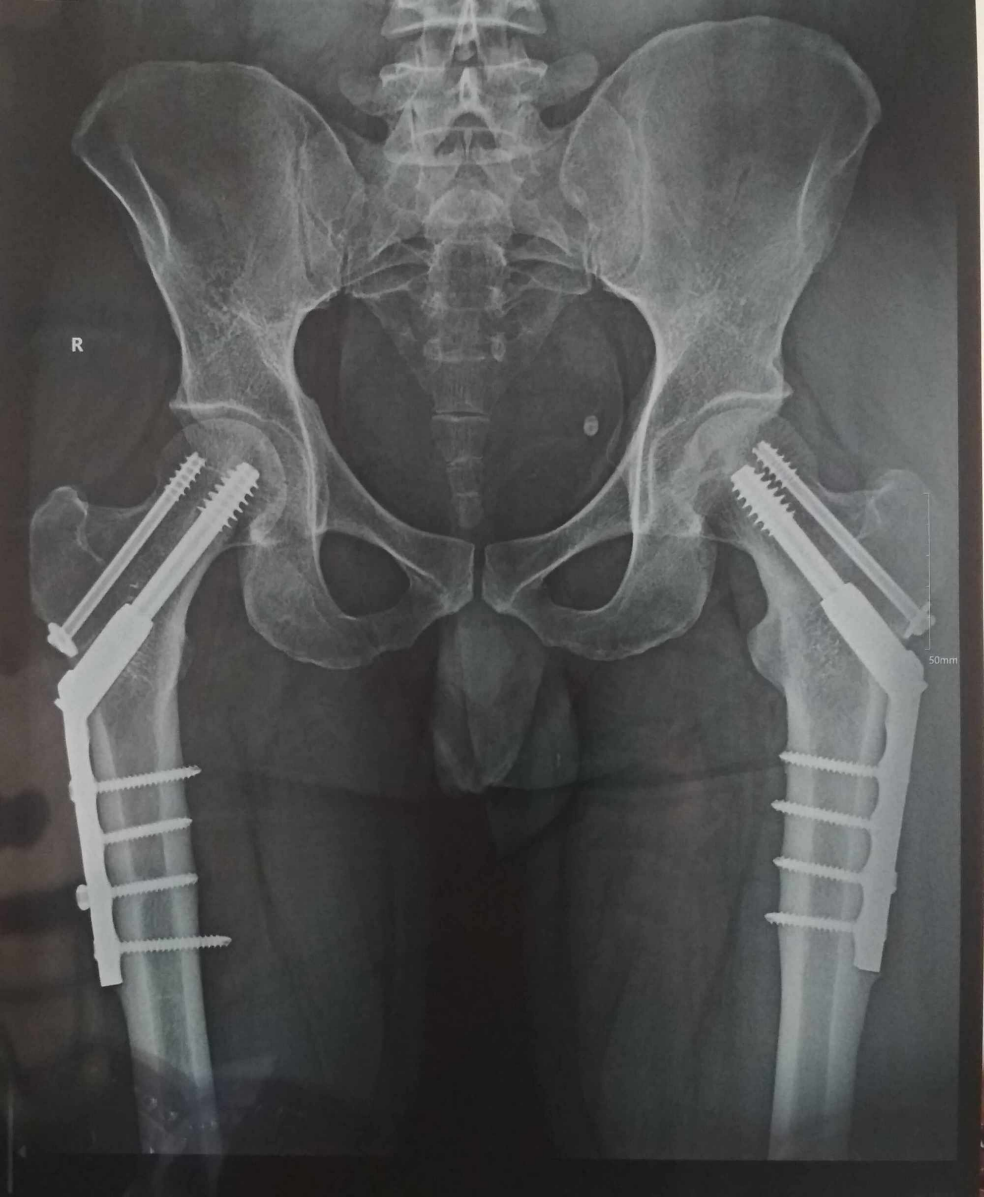
Radiograph at two years showing well united fractures without any complications.

**Figure 5:**
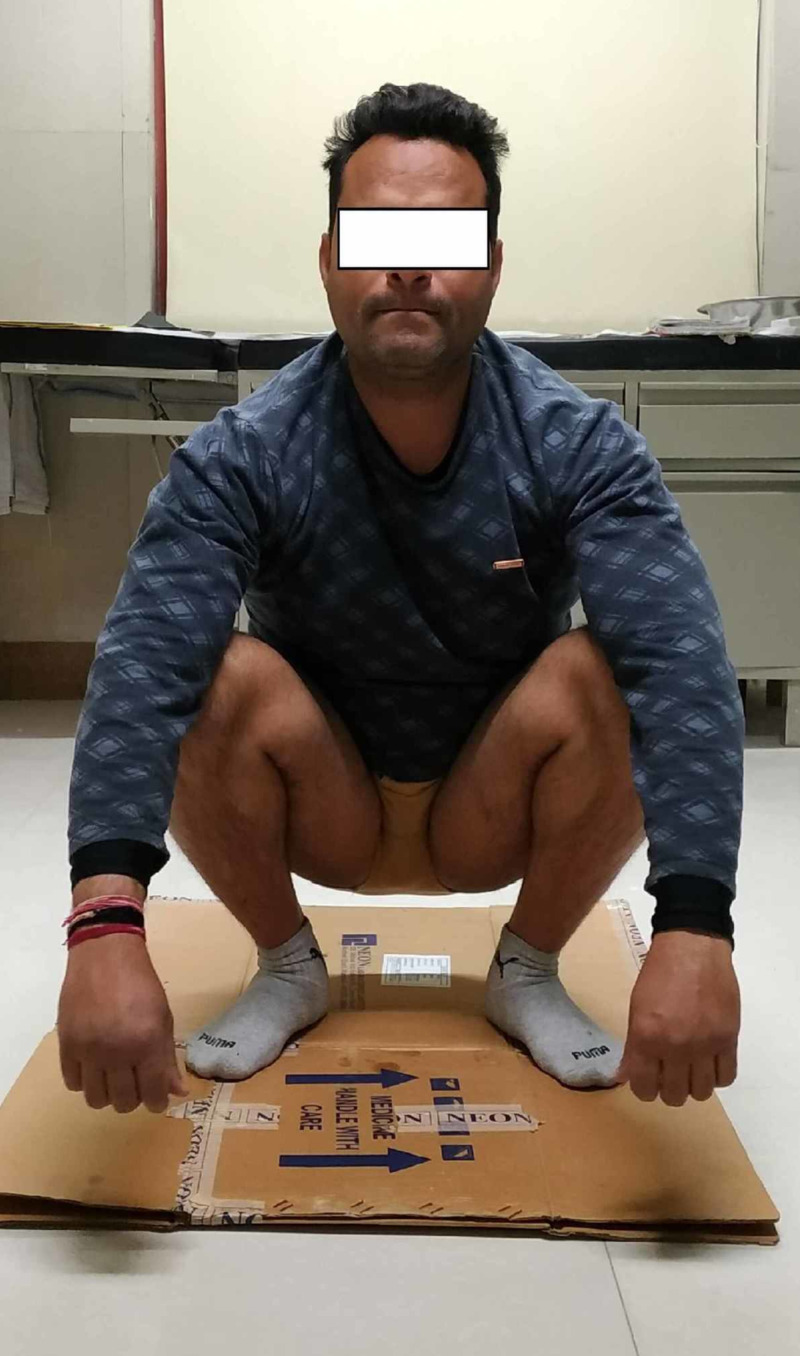
Clinical photograph demonstrating full function at both hip joints.

Post-operatively, patient was kept non-weight-bearing for six weeks, and then gradual partial weight-bearing was started. He was able to walk full weight-bearing at three months. At latest follow-up at two years, the patient is asymptomatic and has full function of both hips (Figures 4, 5).

## Discussion

Stress fracture occurs as a result of inability of bone to withstand repetitive subthreshold force causing it to break partially or completely. It is of two types: fatigue fracture and insufficiency fracture. The “fatigue fracture” results when the normal bone is subjected to abnormal stress, whereas “insufficiency fracture” results when abnormal bone which is lacking elastic resistance is subjected to normal stresses.

Femoral neck stress fractures (FNSF) account for 5% cases. Various risk factors have been described, which include low bone mineral density, abnormal proximal femoral anatomy, corticosteroid use, renal osteodystrophy, osteomalacia, and strenuous physical activity as in athletes and military recruits. FNSF are very rarely seen in healthy, young non-athletic individuals, and the involvement of bilateral hips is even rarer with only a few cases reported in the literature. Most reports of bilateral stress fracture are caused by bone insufficiency and have been seen in either elderly patients or patients with vigorous activity (Table 1).

**Table 1:** Review of existing literature about FNSFs. FNSF, Femoral neck stress fractures; THR, total hip replacement; DHS, dynamic hip screw.

Authors	Study type	Age and occupation	FNSF type	Treatment	Result
de Oliviera et al. [4]	Case Report	43 years, non-athlete, occupation not specified	Bilateral compression type, undisplaced	Bilateral osteosynthesis with two cannulated screws	Long-term follow-up not specified
Mahajan et al. [5]	Case Report	14 years athlete	Right complete with sclerosis over tension side, left side compression type	Conservative	Symptom-free at three months, long-term not specified
Bailie and Lamprecht [6]	Case Report	15 years athlete	Bilateral compression type	Conservative	Followed for last two years, no issues, full return to athletics in one year
Xiaozuo et al. [7]	Case Report	59 years, occupation not specified	Right side compression type, left side displaced	Right conservative, left THR	Right side non-union, symptom-free in one year
Naik et al. [8]	Case series of seven fractures	38-48 years, manual laborers	Compression type - 3, displaced - 3, tension type - 1	Bilateral fixation	Good outcome at long-term
Naranje et al. [9]	Case Report	43 years, military recruit	Right compression type, left displaced	Bilateral osteosynthesis with cannulated screws	Asymptomatic and active at one-year follow-up
Kanwat et al. [10]	Case Report	50-year-old housewife	Right - undisplaced, left - displaced	Right - cannulated screws, left - THR	Asymptomatic at one year
Our study	Case Report	36 years, non-athlete	Bilateral compression type	Bilateral DHS	Full weight-bearing at three months, asymptomatic at two years

A study by Naik et al. reported that repeated vigorous activity results in abnormal stress at hip, which could lead to stress fracture in non-athletes with normal bone [8]. Another theory is that repeated loading on the hip abductors causes muscle fatigue producing a compensatory gait that alters the forces at the hip and results in stress fracture of the femoral neck [11,12].

One interesting history in our patient was that he had taken 10 injections of nandrolone over three months' duration. Nandrolone has been known to increase bone and muscle mass in senile and postmenopausal osteoporosis [13]. Some studies have associated the anabolic action of nandrolone with the increased production of insulin-like growth factor 1 (IGF-I) [14]. However, our patient had no relief in pain with the above injections, which explains that the cause was not related to poor bone density.

The earliest and the most frequent symptom is anterior groin pain. However, up to 75% of occult hip fractures are missed or misdiagnosed by clinical examination alone [15]. Radiographs in early cases are usually normal as can be seen in our case where the fracture was not seen on right side on x-ray. It usually takes around three weeks before any changes are evident on the x-rays and that too are picked up in just 10%-29% cases only [16]. Both MRI and bone scan are sensitive investigations for early diagnosis of these stress fractures. However, Technetium-99 bone scan lacks specificity, whereas MRI is 100% specific in differentiating from tumors and infection, therefore considered as the investigation of choice. In our case, MRI helped in confirmation of diagnosis on the left side and detected the incomplete fracture on the right side.

FNSF are difficult to treat. They can be easily missed initially and can later present with complete displacement of the fracture or osteonecrosis of the femoral head. The undisplaced fracture presents a diagnostic challenge for the clinicians. The study by Johansson et al. reported an average delay of 14 weeks in diagnosis, which can result in displacement to occur in a stress fracture [17]. In young and active individuals with bilateral stress fracture of the femoral neck, even if undisplaced, surgical treatment by stable fixation with early return to physical activity is recommended [18]. The conservative treatment risks displacement of the fracture, which can result in poor outcome with 60% risk of non-union and 30% risk of avascular necrosis [19]. That is why in our case, even though fracture was undisplaced on right side, the patient was treated surgically with osteosynthesis using DHS on both sides.

## Conclusions

In conclusion, stress fracture of femoral neck in a healthy, young non-athletic adult is rare, and bilateral affection is even rarer. These fractures are very commonly missed; so a very high index of suspicion is required in young patients presenting with groin pain without any significant history of trauma. The radiographs must be supplemented with MRI if there is any mismatch in the clinical symptoms and x-ray. Early diagnosis and intervention are of utmost importance for a favorable outcome.

## References

[1] Fullerton LR Jr, Snowdy HA (1988). Femoral neck stress fractures. Am J Sports Med.

[2] Clough TM (2002). Femoral neck stress fracture: the importance of clinical suspicion and early review. Br J Sports Med.

[3] Biz C, Berizzi A, Crimì A, Marcato C, Trovarelli G, Ruggieri P (2017). Management and treatment of femoral neck stress fractures in recreational runners: a report of four cases and review of the literature. Acta Biomed.

[4] de Oliveira US, Labronici PJ, Neto AJ, Nishimi AY, Pires RES, Silva LHP (2016). Bilateral stress fracture of femoral neck in non-athlete-case report. Rev Bras Ortop.

[5] Mahajan A (2017). Simultaneous bilateral femoral neck stress fractures in a young female runner: a case report and review of literature. Saudi J Sports Med.

[6] Bailie DS, Lamprecht DE (2001). Bilateral femoral neck stress fractures in an adolescent male runner: a case report. Am J Sports Med.

[7] Xiaozuo Z, Kai K, Jiangtao D, Shijun G (2016). Bilateral stress fractures of the femoral neck in adults: a case report. Int J Clin Exp Med.

[8] Naik MA, Sujir P, Tripathy SK, Vijayan S, Hameed S, Rao SK (2013). Bilateral stress fractures of femoral neck in non-athletes: a report of four cases. Chin J Traumatol.

[9] Naranje S, Sezo N, Trikha V, Kancherla R, Rijal L, Jha R (2012). Simultaneous bilateral femoral neck stress fractures in a young military cadet: a rare case report. Eur J Orthop Surg Traumatol.

[10] Kanwat H, Mittal S, Trikha V, Malhotra R (2019). Unusual bilateral neck of femur stress fracture in a healthy, non-athletic individual - a case report and literature review. J Orthop Case Rep.

[11] Markey KL (1987). Stress fractures. Clin Sports Med.

[12] Devas MB (1965). Stress fractures of the femoral neck. J Bone Joint Surg Br.

[13] Frisoli A, Chaves PHM, Pinheiro MM, Szejnfeld VL (2005). The effect of nandrolone decanoate on bone mineral density, muscle mass, and hemoglobin levels in elderly women with osteoporosis: a double-blind, randomized, placebo-controlled clinical trial. J Gerontol A Biol Sci Med Sci.

[14] Boonen S, Broos P, Dequeker J, Bouillon R (1997). The prevention or treatment of age-related osteoporosis in the elderly by systemic recombinant growth factor therapy (rhIGF-I or rhTGFβ): a perspective. J Intern Med.

[15] Robertson GA, Wood AM (2017). Femoral neck stress fractures in sport: a current concepts review. Sports Med Int Open.

[16] Ichikawa J, Amano R, Haro H, Sato E, Koyama K, Hamada Y (2008). Fatigue fracture of the bilateral femoral neck in the elderly. Orthopedics.

[17] Johansson C, Ekenman I, Törnkvist H, Eriksson E (1990). Stress fractures of the femoral neck in athletes. The consequence of a delay in diagnosis. Am J Sports Med.

[18] Jones BH, Thacker SB, Gilchrist J, Kimsey CD, Sosin DM (2002). Prevention of lower extremity stress fractures in athletes and soldiers: a systematic review. Epidemiol Rev.

[19] Diwanji SR, Kong IK, Cho SG, Seon JK, Yoon TR (2007). Displaced stress fracture of the femoral neck treated by valgus subtrochanteric osteotomy: 2 case studies. Am J Sports Med.

